# Study on the Influence of Calcined Underground Ant Nest Powder on the Durability of Concrete

**DOI:** 10.3390/ma13092119

**Published:** 2020-05-02

**Authors:** Wei Zhou, Peng Zhu, Wenjun Qu, Wu Yao, Shengji Wu

**Affiliations:** 1 Key Laboratory of Advanced Civil Engineering Materials of Ministry of Education, School of Materials Science and Engineering, Tongji University, Shanghai 200092, China; weizhou_tj@tongji.edu.cn (W.Z.); yaowuk@tongji.edu.cn (W.Y.); 2 Department of Structural Engineering, Tongji University, Shanghai 200092, China; quwenjun.tj@tongji.edu.cn (W.Q.); wushengji@tongji.edu.cn (S.W.); 3 Key Laboratory of Performance Evolution and Control for Engineering Structures, Tongji University, Ministry of Education, Shanghai 200092, China

**Keywords:** underground ant nests, calcined ant nest clay powder (CANCP), durability, chloride penetration resistance, carbonization, freeze–thaw resistance

## Abstract

Ants have strict requirements on the building materials of the nest, such as the size, weight, luster and color of soil particles. The soil of underground ant nests is composed of clay particles cemented together to form a hard brick-like material. The ant nest powder shows pozzolanic activity after calcination, which can meet the requirements for active admixture of concrete. Under the standard curing condition, the influence of calcined ant nest clay powder (CANCP) on the durability of concrete is evaluated by chloride penetration resistance, carbonization resistance and freeze–thaw resistance, and the influence of the powder content is investigated. The results show that when the content of CANCP is less than 10%, the chloride penetration resistance of concrete increases with content of CANCP. In the early stage of carbonation, the greater the content of CANCP, the higher the carbonization rate of concrete. In the middle and later stage of carbonation, the carbonation rate of CANCP concrete is significantly lower than that in the early stage, and the carbonation depth is linearly related to the carbonation time. When the content of CANCP is less than 20%, the freeze–thaw resistance of CANCP concrete is better than that of the reference concrete.

## 1. Introduction

Ants have strict requirements on the building materials of the nest, such as the size, weight, luster and color of soil particles. The underground ant nest is generally composed of soil and gravel, and is mixed with organic substances such as dead plant bodies and ant secretion, and the clay content is large. Compared with the common clay around the ant nest, its physical and chemical properties have changed greatly. The soil of underground ant nests is composed of clay particles cemented together to form a hard brick-like material. Most of the natural clays have pozzolanic activity after calcination [1]. At present, the pozzolanic active substances that can partially replace cement and meet the requirements of concrete mineral admixture are as follows: natural products such as pozzolan and zeolite; solid wastes and industrial by-products such as slag, fly ash, silica fume, rice husk ash and clay brick [2,3,4,5,6], etc.; low energy products, such as calcined clay [7,8,9] and calcined termite clay [1].

The calcined ant nest clay powder (CANCP) can partially replace cement and the associated influence on the compressive strength of concrete has been studied [10]. After calcination, the powder of ant nest soil has the properties of pozzolanic ash, which meets the requirements of concrete active admixture, and CANCP can improve the strength of mortar and concrete. Concrete durability is the ability of concrete to resist various physical and chemical actions such as climate change, chemical erosion, abrasion under the actual real environment. It is directly related to the safety and performance of buildings, and it is an important standard to measure the quality of concrete. In order to complete the study on the influence of CANCP on concrete, the influence of CANCP on the durability of concrete is evaluated through experiments in this paper, including chloride penetration resistance, carbonization resistance and freeze–thaw resistance.

## 2. Materials and Methods

PO 42.5 ordinary Portland cement was used [11], and the physical and mechanical properties are listed in Table 1. The basic material properties of natural river sand and stones used in this test are shown in Table 2 and Table 3. The DY-106 polycarboxylate high-performance water-reducing agent was selected, and the performance parameters are shown in Table 4.

A standard mix proportion of design strength of C50 was used as reference concrete (CT for short) in this test, and the calculated mix proportions were shown in Table 5. The CANCP was used to replace cement in proportions of 5%, 10%, 15% and 20% to study the effect on concrete durability properties. By adjusting the dosage of water-reducing agent, concrete slump was controlled near 200 mm.

### 2.1. Properties of CANCP

**Figure 1:**
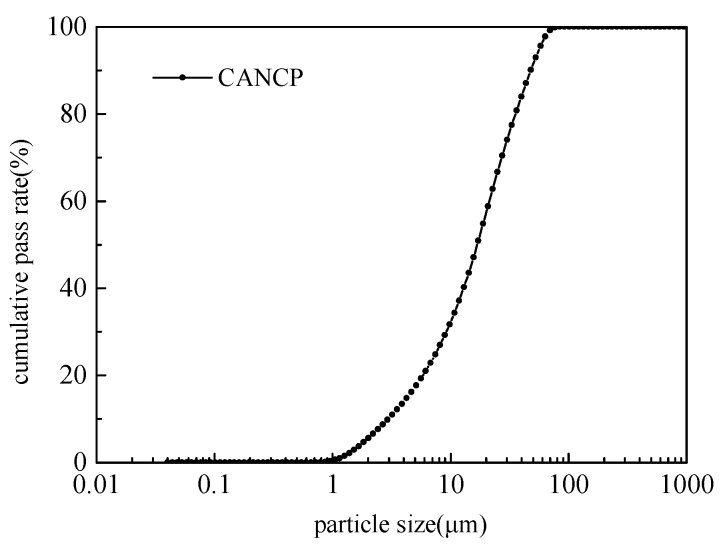
Particle size distribution curves of CANCP.

CANCP was obtained from the underground nest soil of iridomyrmex anceps after calcination at 800 °C for 2 h and grinding for 60 min by SM-500 ball crusher [10]. The physical properties of CANCP are shown in Table 6. The margin of 45 μm square hole sieves of CANCP was 16.58% and 16.25% respectively after two screening tests, which is close to the 12% requirement for first-grade fly ash [12]. The particle size distribution of CANCP is continuous, and the proportion of particles with size of 1–10 μm is large. The particle size distribution curve of CANCP is shown in Figure 1.

The total content of SiO_2_, Al_2_O_3_ and Fe_2_O_3_ in CANCP reached 88.76% which is greater than the 70% specified in the ASTM (American Society of Testing Materials) standard for concrete mineral admixtures [13]. The chemical composition of CANCP is shown in Table 7.

### 2.2. Test Methods

#### 2.2.1. Chloride Penetration Resistance

**Figure 2:**
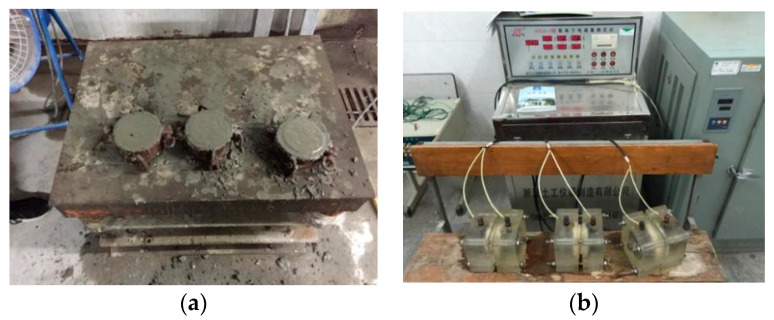
(**a**) Vibration forming and (**b**) test process of the specimens.

In this test, the electric flux method of ASTM C1202 [14] was used to evaluate the chloride penetration resistance of concrete with different content of CANCP. Five groups (three specimens in each group) of 100 mm diameter and 50 mm thick cylinder concrete specimens with 0%, 5%, 10%, 15% and 20% of CANCP were made respectively (Table 5). The chloride penetration resistance of CANCP concrete was tested by concrete electric flux meter of type NEL-PEU. Twenty-four hours before the test, the specimens were sealed on the side with paraffin, and then vacuum water saturation of cylinder specimens were tested at the curing age by concrete intelligent vacuum water saturator of type NEL-VJH. Each specimen was split along the axis after the electric flux test, and 0.1 mol/l silver nitrate solution was sprayed on the section. The penetration depth X_D_ of chloride ion was measured. The vibration forming and test process of the specimens are shown in Figure 2.

#### 2.2.2. Carbonation Resistance

In this test, the method of GB/T 50082-2009 [15] was used to evaluate the carbonation performance of concrete with different content of CANCP. Three groups (three in each group) of 100 mm × 100 mm × 400 mm prism concrete specimens with 0%, 10% and 20% of CANCP were made respectively (Table 5).

Standard curing was adopted, and the specimens were taken out from the standard curing room two days before the test, and then they were dried for 48 h in the electric air blast drying oven at 60 °C After drying, except one side of the specimen, the other three sides were sealed with heated paraffin. On the exposed side, parallel lines were drawn with a pencil at a distance of 10 mm along the length direction as the measurement point of the predetermined carbonation depth. HTX-12X microcomputer concrete carbonation test chamber was used in the test, and the test was carried out under the conditions of CO_2_ concentration (20% ± 3%), relative humidity (70% ± 5%), and temperature (20 ± 2 °C).

**Figure 3:**
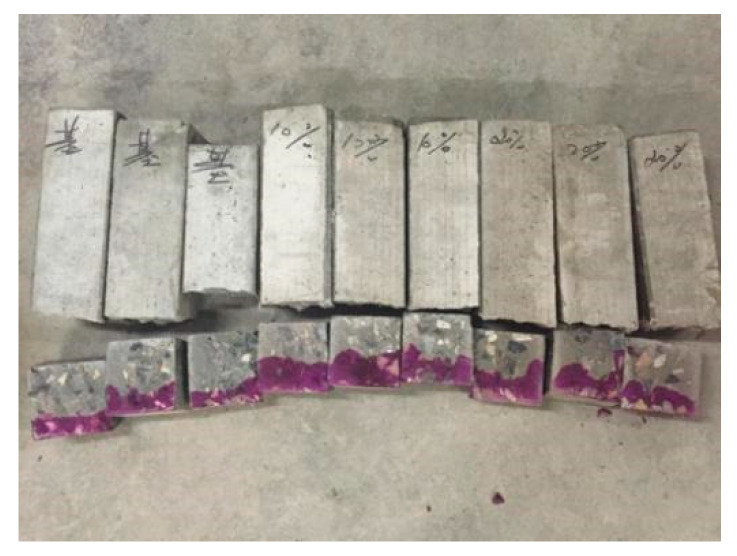
Determination carbonation depth of specimens.

The specimens were taken out when the carbonization process reached 3, 7, 14 and 28 d, and the splitting method was used on the pressure testing machine to start the fracture from 50 mm at one end. After the fracture, the cross section was sealed with paraffin again, and the remaining specimen was put into the carbonization test chamber to continue carbonization until the next carbonization age. The residual powder was removed on the section of the specimen obtained by splitting, and 1% phenolphthalein reagent was sprayed immediately on the cross section. After 30 s, the carbonation depth of 10 measuring points was measured respectively. The average value of carbonation depth at each test point was calculated, as the carbonation depth of the specimen, and the calculation accuracy was 0.001 mm. The determination carbonation depth of specimens is shown in Figure 3.

#### 2.2.3. Freeze–Thaw Resistance

First, 100 mm × 100 mm × 400 mm prism specimen was adopted for the quick freeze–thaw test [16]. The specimens were cured in the standard curing room, and they were taken out from the curing room when the curing age was 24 d. Then, the specimens were soaked in water at (20 ± 2 °C) for 4 d, and the freeze–thaw test was carried out in the full-automatic anti-freeze instrument. The freeze–thaw time was in accordance with procedure A of ASTM C666 [16], i.e., 2–5 h of each freeze–thaw cycle (4 h in this test), the temperature was reduced from 4 to −18 °C and increased from −18 to 4 °C, ensuring that 25% of the time was used for thawing. Three groups (three in each group) of 100 mm × 100 mm × 400 mm prism concrete specimens with 0%, 10% and 20% of CANCP were made respectively (Table 5).

**Figure 4:**
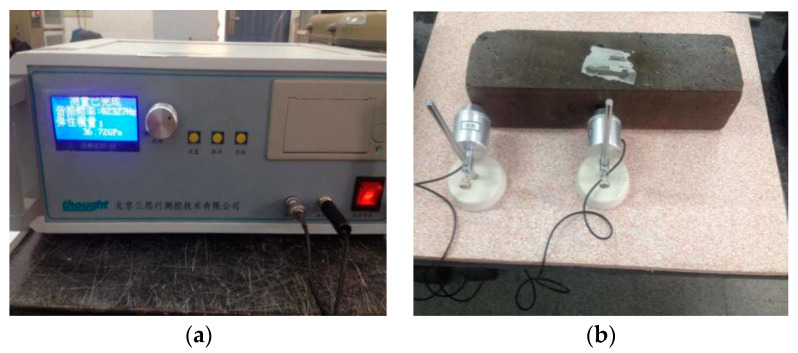
(**a**)Transverse fundamental frequency measurement and (**b**) position of measuring point after freeze–thaw cycle.

The mass loss rate ΔW and the relative dynamic elasticity modulus reduction rate ΔD are taken as the indexes to evaluate the freeze–thaw resistance performance of concrete. The mass and transverse fundamental frequency of the specimens were measured every 25 freeze–thaw cycles, as shown in Figure 4.

## 3. Results

### 3.1. Influence of CANCAP on the Chloride Penetration Resistance of Concrete

#### 3.1.1. Influence of CANCP on Each Index of Electric Flux

According to the electric flux method of ASTM C1202 [14], the 6 h electric flux value Q, the initial current value I_0_ and the chloride ion penetration depth value X_D_ were measured, and the results are shown in Table 8.

It can be seen from the test results that the chloride penetration resistance of concrete increases first and then decreases after adding CANCP. When the content of CANCP is less than 10%, the chloride penetration resistance of concrete increases with the increase of the content of CANCP, and the electric flux of concrete containing 5% and 10% CANCP is 1310.1C and 998.9C, which is 8.8% and 30.4% lower than that of the reference concrete CT. When the content of CANCP is more than 10%, the chloride penetration resistance of concrete decreases with the increase of the content of CANCP, and the electric flux of concrete containing 15% and 20% CANCP is 1639.5C and 2207.1C, which is 14.2% and 53.7% higher than that of the reference concrete CT. On the contrary, the initial current value and chloride penetration depth value of concrete first decreased and then increased after adding CANCP. When the content of CANCP is less than 10%, the initial current value and chloride penetration depth of concrete decrease with the increase of the content of CANCP; when the content of CANCP is more than 10%, the initial current value and chloride penetration depth of concrete increase with the increase of the content of CANCP.

#### 3.1.2. Correlation Analysis of Initial Current and 6 h Electric Flux

**Figure 5:**
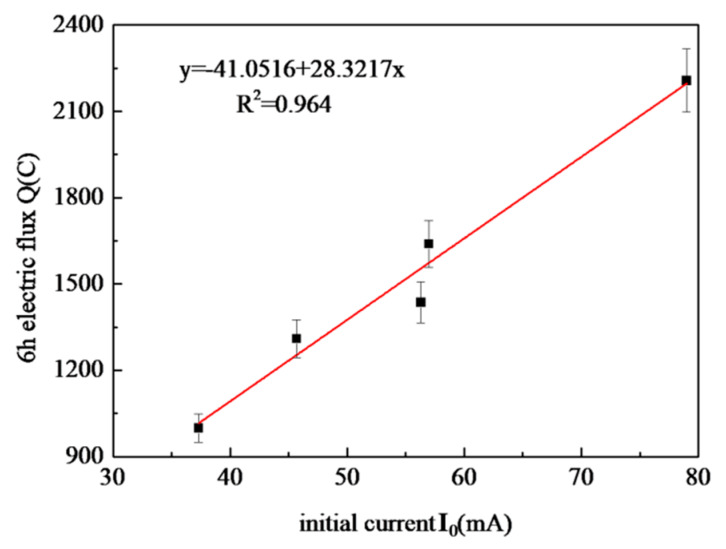
Correlation between initial current and 6 h electric flux.

Feldman believes that there is a good linear correlation between the initial current or conductivity and the 6 h electric flux for the same type of concrete. Therefore, it can be considered to provide a fast and convenient evaluation method for the chloride penetration resistance of concrete by testing the initial current or conductivity [17]. The linear correlation analysis of initial current and 6 h electric flux is shown in Figure 5. It can be seen from the figure that the correlation index R^2^ is 0.964, and there is a good linear correlation between the initial current and 6 h electric flux. Therefore, the initial current can be used to calculate the electric flux to accelerate the test efficiency. The 28 d initial current can be used as the evaluation index to accelerate the detection of the chloride penetration resistance of CANCP concrete.

#### 3.1.3. Correlation Analysis of Chloride Penetration Depth and 6 h Electric Flux

**Figure 6:**
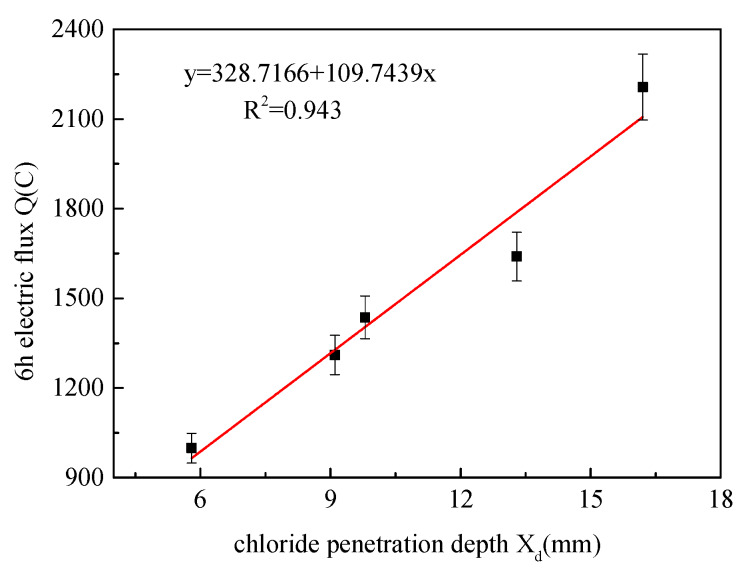
Correlation between chloride penetration depth and 6 h electric flux.

The linear correlation analysis of chloride penetration depth and 6 h electric flux is shown in Figure 6. It can be seen from the figure that the correlation index R^2^ is 0.943, and there is a good linear correlation between chloride penetration depth and 6 h electric flux. Therefore, the penetration depth X_D_ can be calculated by measuring the electric flux Q, and then the permeability coefficient of chloride ion in the specimen can be obtained according to Nernst–Planck equation.

### 3.2. Influence of CANCAP on the Carbonization Resistance of Concrete

According to JGJ/T 193-2009 [18], the carbonation resistance of concrete was evaluated according to the carbonation age of 28 d, as shown in Table 9.

**Figure 7:**
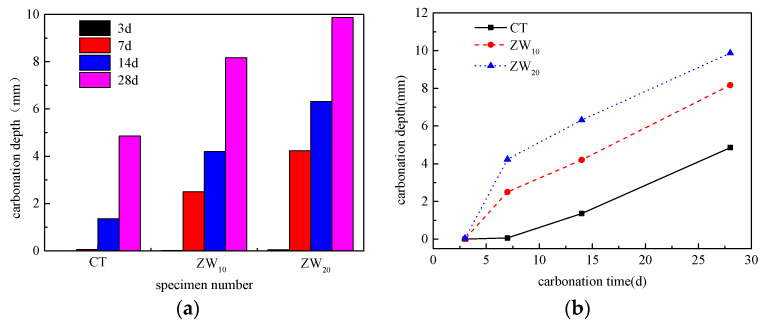
(**a**) Carbonation depth histogram and (**b**) carbonation depth-time curve of concrete with different content of CANCP.

The carbonation depth histogram and the carbonation depth-time curve of concrete with different content of CANCP are shown in Figure 7.

It can be seen from the histogram that the carbonation depth of the concrete with CANCP in each curing age is greater than that of the reference concrete. With the increase of the content of CANCP, the carbonation depth gradually increases. The carbonation time is 28 days, and the carbonation depth of concrete with 20% of CANCP is 9.863 mm, which is more than twice of that of concrete without CANCP. As shown in Table 8, when the content of CANCP is not more than 20%, the carbonation resistance grade of concrete is T-IV, that is, when the content is not more than 20%, the carbonation resistance performance grade of concrete will not be impacted by CANCP. It can be seen from Figure 7b that the carbonation depth of concrete with different content of CANCP increases gradually with the carbonation time. In the early stage of carbonation, the higher the content of CANCP is, the higher the carbonation rate is, and the influence of CANCP on the early carbonation performance of concrete is obvious. In the middle and later stage of carbonation, the carbonation rate of CANCP concrete is significantly lower than that of the early stage, and the carbonation depth is basically linearly related to the carbonation time.

### 3.3. Influence of CANCAP on the Freeze–Thaw Resistance of Concrete

**Figure 8:**
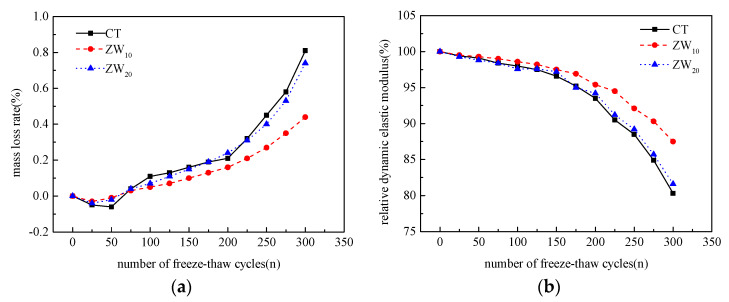
(**a**) Mass loss rate and (**b**) relative dynamic elastic modulus curve of different content of CANCP with the number of freeze–thaw cycles.

The mass loss rate and relative dynamic elastic modulus curve of different content of CANCP with the number of freeze–thaw cycles, as shown in Figure 8.

#### 3.3.1. Influence of Different Content of CANCP on the Mass Loss Rate of Concrete during Freeze–Thaw Cycle

It can be seen from Figure 8 that the mass of concrete with different content of CANCP has a certain degree of increase at the early stage of the freeze–thaw cycle. This is because the pumice aggregate in concrete is a honeycomb structure, with a large number of open pores on the surface and strong water absorption capacity, which leads to the water absorption of concrete greater than the surface peeling off, thus increasing the mass. In the middle and later period of the freeze–thaw cycle (after 50 cycles), the mass loss of concrete with different content of CANCP increases with the increase of freeze–thaw cycles. After 300 cycles of freeze–thaw, the mass loss rate of the reference group without CANCP is the largest, reaching 0.81%. The mass loss rate of the group with 10% of CANCP is 0.44%, which is significantly lower than that of the reference group, and the mass loss of concrete can be reduced by adding CANCP. The mass loss rate of the group with 20% of CANCP is 0.74%, which is slightly lower than that of the reference group without CANCP, and the mass loss rate of each cycle is close to that of the reference group without CANCP, and it shows that the improvement effect of further increasing the content of CANCP on the mass loss rate of concrete mass is not obvious, and even the mass loss rate of the partial freeze–thaw cycle stage is reduced.

#### 3.3.2. Influence of Different Content of CANCP on Relative Dynamic Modulus of Elasticity of Concrete during Freeze–Thaw Cycle

The dynamic modulus of elasticity is closely related to the internal structure of materials, that is, the dynamic modulus of elasticity of concrete is more sensitive to the internal structure damage. Under the action of the freeze–thaw cycle, the internal structure of the concrete gradually changes from compact to loose, and the concrete itself inevitably has some original microcracks and defects. With the increase of freeze–thaw cycle times, the microcracks and defects in the concrete continue to expand, the section strength gradually decreases, the internal microstructure of concrete is disturbed, resulting in the decrease of dynamic elastic modulus of the concrete.

The decrease of dynamic modulus of elasticity of each content is not obvious in the early stage of the freeze–thaw cycle (before 100 cycles), and the accelerated failure appears in the middle and later stage of the test. The accelerated failure occurred after 150 freeze–thaw cycles for the reference group without CANCP and the group with 20% of CANCP, while the accelerated failure occurred after 225 cycles for the group with 10% of CANCP. The concrete with 10% of CANCP demonstrated the best freeze–thaw resistance.

#### 3.3.3. Freeze–Thaw Resistance Durability of Concrete with Different Content of CANCP

The freeze–thaw resistance durability index K_b_ is used to estimate the freeze–thaw resistance of concrete:

(1) $$K_{b}=\left(P\times N\right)/300$$

In the formula: P is the relative dynamic elastic modulus of concrete after n freeze–thaw cycles; N is the number of freeze–thaw cycles that the concrete can withstand.

The calculated freeze–thaw resistance durability index of CANCP concrete is shown in Table 10. The freeze–thaw resistance durability index greater than 60 is considered to be good, that between 40 and 60 is normal, and that less than 40 is poor [19]. It can be seen from Table 10 that the freeze–thaw resistance of concrete is improved by CANCP. When the CANCP content is 10% and 20%, the durability index is 87.5 and 81.6, and the freeze–thaw resistance of concrete is high.

## 4. Discussion

### 4.1. Reason of Improved the Chloride Penetration Resistance of Concrete by CANCP

**Figure 9:**
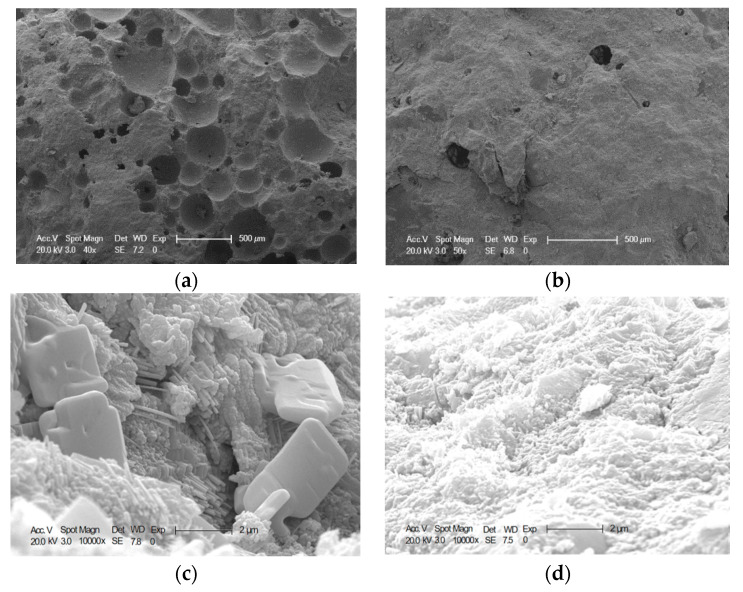
(**a**,**c**) 40×, 10000× ordinary concrete (28 days curing) and (**b**,**d**) 50×, 10000× 10% CANCP concrete (28 days curing) SEM micrographs.

When the content of CANCP is not more than 10%, it can effectively improve the chloride penetration resistance of concrete. The reasons are listed below:

1. SiO_2_ and Al_2_O_3_ in CANCP can generate more hydrated calcium silicate and aluminum silicate with Ca(OH)_2_, which can increase the binding capacity of concrete to chloride ions and reduce the content of free chloride ions [20].
2. Micro filling effect and micro aggregate effect [21]. The fine particles of CANCP are filled between the cement particles, reducing the void and capillary ratio, and improving the microstructure of concrete, as can be seen from Figure 9a,b.
3. Pozzolanic activity and pozzolanic effect of CANCP promotes the secondary hydration reaction of cement, resulting in a large number of C-S-H gels. A large amount of Ca(OH)_2_ is consumed in the hydrated cement paste, and C-S-H gel is increased during the process of pozzolanic reaction. The pozzolanic effect of CANCP can transform Ca(OH)_2_ to C-S-H gel, and fill between cement hydration products, which can effectively improve the performance of concrete. Meanwhile, the pozzolanic C-S-H gel with low Ca/Si ratio will produce when CANCP has second pozzolanic reactions with traditional C-S-H gel in the hydrated cement paste, and the pozzolanic C-S-H gel is relatively stable which can improve the performance of the traditional C-S-H gel to improve the performance of the concrete. The addition of CANCP can improve the microstructure of the concrete, reduce the porosity, refine the pore size and make the concrete more dense, and all of these increase the diffusion resistance of the concrete to chloride penetration [22]. It can be seen form Figure 9c,d that there is no content of Ca(OH)_2_ in concrete containing 10% CANCP, and it shows that concrete containing 10% CANCP has a high degree of hydration; C-S-H gel in concrete with 10% of CANCP is more dense and clustered to a higher degree than ordinary concrete.

### 4.2. Reason of Accelerated Carbonation Resistance of Concrete by CANCP

The secondary hydration reaction of the active components SiO_2_, Al_2_O_3_ and Fe_2_O_3_ in CANCP with the main products of cement hydration is as follows [23]:

(2) $$CaO+H_{2}O=Ca{\left(OH\right)}_{2}$$

(3) $$2SiO_{2}+3Ca{\left(OH\right)}_{2}=3CaO\cdot 2SiO_{2}\cdot 3H_{2}O$$

(4) $$3CaO\cdot 2SiO_{2}\cdot 3H_{2}O+xSiO_{2}+yH_{2}O=z\left[\left(0.8\sim 1.5\right)CaO\cdot SiO_{2}\cdot qH_{2}O\right]$$

(5) $$Al_{2}O_{3}+Fe_{2}O_{3}+8Ca{\left(OH\right)}_{2}+18H_{2}O=8CaO\cdot Al_{2}O_{3}\cdot Fe_{2}O_{3}\cdot 26H_{2}O$$

CANCP has active ingredients, and its secondary hydration requires consumption of Ca(OH)_2_, resulting in C-S-H gel and C-A-H gel with low calcium–silicon ratio. At the same time, the active silicon in CANCP will react directly with the C-S-H gel generated from the cement to form C-S-H gel with low calcium–silicon ratio [24,25]. Although the addition of CANCP can optimize the microstructure of concrete to a certain extent, the increase of CANCP results in the decrease of cement content per unit volume. At the same time, the active SiO_2_ and Al_2_O_3_ in the admixture react with Ca(OH)_2_ generated by secondary hydration of the cement clinker, which reduces the alkali reserve in the cementitious paste [26]. When the content of CANCP is large, the alkali reserve in the paste may be exhausted. This results in the poor carbonation resistance of concrete and accelerates the carbonation process.

The carbonization rate of CANCP concrete at the early stage of carbonization is much larger than that in the middle and later stage of carbonation. This is due to the large number of pores in cementitious material in the early stage. In the middle and later stage, the pozzolanic reaction occurred, and the products of C-S-H gel and carbonization reaction product CaCO_3_ have filling effect. The internal pore structure of cementitious materials is improved, resulting in slow carbonation reaction.

### 4.3. Reason of Improved the Freeze–Thaw Resistance of Concrete by CANCP

The pozzolanic effect by CANCP can result in the formation of low alkaline calcium silicate hydrate with high strength, increasing the amount of hydration cementitious material in concrete, improving the ability of the traditional C-S-H gel, thereby improving the performance of hardened cement paste and improving the freeze–thaw resistance property of concrete. At the same time, micro filling effect by CANCP can result in denser paste, and improved freeze–thaw resistance property [27,28].

The content of CANCP should be controlled at a proper proportion, and the best proportion is 10%. When the content is more than 20%, the hydration of CANCP will not be complete. At the same time, non-active components in CANCP remain in the concrete to form more pores which increases the porosity of concrete. Under the action of the freeze–thaw cycle, micro cracks will be formed in the concrete, which lead to the deterioration of the internal structure of the concrete.

## 5. Conclusions

This paper attempts to prove that CANCP can be used as a mineral admixture to improve the durability of concrete. Based on the experimental tests and the data analyses, the following conclusions can be drawn:

1. When the content of CANCP is less than 10%, the initial current value and chloride penetration depth of concrete decrease with the increase of the content of CANCP and it can effectively improve the chloride penetration resistance of concrete.
2. The addition of CANCP can accelerate the carbonation process of concrete. When the CANCP content is not more than 20%, the carbonation resistance grade of concrete is not impacted.
3. When the content of CANCP is less than 10%, the concrete with CANCP can still keep the internal structure compact under the action of freeze–thaw and it can effectively improve the freeze–thaw resistance of concrete.
4. It is proved that CANCP can be used as green concrete admixture, which provides favorable evidence for the feasibility of CANCP as ecological building materials.

## Figures and Tables

**Table 1:** Physical and mechanical properties of Portland cement.

Fineness (%)	Density (g/cm^3^)	Setting Time (min)		Flexural Strength (MPa)		Compressive Strength (MPa)	
		Initial Setting	Final Setting	3 days	28 days	3 days	28 days
5.7	3.13	150	490	5.5	7.6	24.6	44.3

**Table 2:** Properties of the natural river sand.

Water Content (%)	Sediment Percentage (%)	Apparent Density (kg/m^3^)	Compact Packing Density (kg/m^3^)	Loose Packing Density (kg/m^3^)
2.0	1.5	2570	1760	1650

**Table 3:** Properties of the natural stone.

Water Content (%)	Sediment Percentage (%)	Apparent Density (kg/m^3^)	Compact Packing Density (kg/m^3^)	Loose Packing Density (kg/m^3^)	Crushing Value (%)
0.8	0.9	2700	1430	1650	6.6

**Table 4:** Parameters of the water-reducing agents.

Solid Content (%)	Density	pH	Water-Reducing Rate of Concrete (%)	Fluidity of Cement Paste (mm)	Chloride Ion Content (%)	Total Alkalinity
40.19	1.090	6.0	30.0	285	0.05	1.2

**Table 5:** Mix proportion and slump (kg/m^3^).

Specimen Number	Cement	CANCP	Sand	Stone	Water	Water Reducer	Slump
CT	486	0	741	1023	170	0.96	220
ZW_5_	461.7	24.3	741	1023	170	0.97	220
ZW_10_	437.4	48.6	741	1023	170	1.22	240
ZW_15_	413.1	72.9	741	1023	170	1.99	220
ZW_20_	388.8	97.2	741	1023	170	2.02	220

**Table 6:** Physical properties of CANCP.

Loss on Ignition (%)	Specific Gravity	Specific Surface Area (m^2^/kg)	Average Particle Size (μm)
22.5	2.66	491	23.35

**Table 7:** Chemical composition of CANCP (%).

Constituent	SiO_2_	Al_2_O_3_	Fe_2_O_3_	CaO	K_2_O	MgO	Na_2_O	SO_3_
CANCP	69.5	14.1	5.16	2.27	2.81	2.24	1.34	0.14

**Table 8:** Six h electric flux value, initial current value and chloride penetration depth value of concrete with different content of CANCP.

Specimen Number	Electric Flux Q/C		Initial Current I_0_/mA		Chloride Penetration Depth X_d_/mm		Electric Flux Evaluation
	Each	Avg	Each	Avg	Each	Avg	
CT	1583.1	1436.1	62	56.3	11.1	9.8	low
	1330.2		51		9.0		
	1395		56		9.2		
ZW_5_	1184.4	1310.1	42	45.7	7.9	9.1	low
	1323.0		44		9.3		
	1422.9		51		10.2		
ZW_10_	1017.8	998.9	38	37.3	6.1	5.8	very low
	970.1		36		5.4		
	1008.8		38		5.9		
ZW_15_	1669.5	1639.5	58	57.0	13.7	13.3	low
	1621.8		56		13.2		
	1627.2		57		12.9		
ZW_20_	2151.9	2207.1	77	79.0	16.1	16.2	medium
	2324.7		81		16.7		
	2144.7		79		15.9		

**Table 9:** Classification of carbonation resistance of concrete.

Grade	T-I	T-II	T-III	T-IV	T-V
Carbonation depth d (mm)	d ≥ 30	20 ≤ d < 30	10 ≤ d < 20	0.1 ≤ d < 10	D < 0.1

**Table 10:** Freeze–thaw resistance durability index of CANCP concrete.

Specimen Number	CT	ZW_10_	ZW_20_
Durability index	80.3	87.5	81.6
